# Molecular characterization of selected multidrug resistant *Pseudomonas* from water distribution systems in southwestern Nigeria

**DOI:** 10.1186/s12941-015-0102-4

**Published:** 2015-09-02

**Authors:** Ayodele T. Adesoji, Adeniyi A. Ogunjobi, Isaac O. Olatoye

**Affiliations:** Department of Biological Sciences, Federal University Dutsin-Ma, Dutsin-Ma, Katsina State Nigeria; Department of Microbiology, University of Ibadan, Ibadan, Nigeria; Department of Veterinary Public Health and Preventive Medicine, University of Ibadan, Ibadan, Oyo State Nigeria; Paul G. Allen School for Global Animal Health, Washington State University, Pullman, WA USA

**Keywords:** *Pseudomonas* spp, Antibiotic resistance genes, Multidrug resistance, Water distribution systems

## Abstract

**Background:**

Persistence of antibiotic resistant bacteria, including multidrug resistant (MDR) pseudomonads, is an important environmental health problem associated with drinking water distribution systems (DWDS) worldwide. There is paucity of data on the molecular characteristics of antibiotic resistance genes and their mode of transfer among pseudomonads from DWDS located in resource-challenged areas such as southwestern Nigeria.

**Methods:**

MDR pseudomonads (n = 22) were selected from a panel of 296 different strains that were isolated from treated and untreated water in six DWDS located across southwest Nigeria. Primarily, the isolated pseudomonads strains were identified by 16S rDNA sequencing and antibiotic-resistance testing was completed using agar breakpoints assays. The final panel of strains of resistant to more than three classes of antibiotics (i.e. MDR), were further characterized by PCR genotyping, Sanger sequencing, and plasmid profiling.

**Results:**

Pseudomonad resistance to gentamicin and streptomycin ranged from 22.7 to 54.6 % while resistance to tetracycline, ceftiofur and sulphamethoxazole ranged from 40.9 to 77.3 %. The most commonly detected antibiotic resistance genes were *tet*(A) (31.8 % of isolates), *sul1* (31.8 %), *bla*_*TEM*_ (40.9 %) and *aph*(*3″*)^*c*^ (36.4 %). Class 1 integron sequences were evident in 27.3 % of isolates and they harbored genes encoding resistance to aminoglycosides (*aadA2*, *aadA1*), trimethoprim (*dfrA15*, *dfr7*) and sulphonamide (*sul1*) while the plasmid ranged between 22 and 130 kb.

**Conclusions:**

*Pseudomonas* spp, isolated from these DWDS possess resistance genes and factors that are of public and environmental health significance. Therefore, has the potential of contributing to the global scourge of resistance genes transfer in human, animals and environments, thereby, useful in the epidemiology of antimicrobial resistance.

## Background

*Pseudomonas* spp, is a ubiquitous and diverse genus of Gram-negative bacteria that can be found in soil, water, decaying vegetation and animals. According to Craun et al. [1], *Pseudomonas* was one of the most frequently identified agents associated with waterborne outbreaks of dermatitis (rash or folliculitis), as well as conjunctivitis, otitis externa and other symptoms from recreational water in the US Pseudomonads are well adapted to survival in the warm temperatures found in whirlpools, hot tubs and indoor pools.

The most important and ubiquitous pathogenic pseudomonad is *P. aeruginosa.* It is medically significant and has a high intrinsic resistance to antibiotics while causing wide spectrum of opportunistic infections [2]. *P. aeruginosa* is best known for chronic lung infections among cystic fibrosis patients [3]. *P. aeruginosa* is also a cause of serious infections among immunocompromised cancer patients, burn patients, catheterized patients, and other hospitalized individuals [2].

Antibiotic resistance genes can be transferred between different microorganisms [4]. Mobile genetic elements such as plasmids, transposons and integrons contribute to *P. aeruginosa* multidrug resistance [5–8]. There is, however, a paucity of information about the molecular basis for antibiotic resistance for environmental *Pseudomonas* in southwestern Nigeria. Hence, we characterized selected multidrug resistant bacteria from water distribution systems from southwestern Nigeria using 16S rDNA sequencing and PCR genotyping.

## Methods

### Bacterial isolates

Independent multidrug resistant *Pseudomonas* isolates (n = 22) were selected between December 2010 and July 2011 from a pool of two hundred and ninety-one different bacteria genera from raw, treated and municipal taps of six selected water distribution systems in Ife, Ede, Asejire, Eleyele, Owena-Ondo and Owena-Idanre, in southwestern Nigeria. Methods for sampling and bacterial isolation were described previously [10]. Isolates were stored in freezing medium at −80 °C in 96-well plates until analysis.

### Description of sample locations

The description of these dams’ water distribution systems selected for this study is in our previous publications [9–11]. However, for clarity of this paper, raw water samples were taken from the untreated water from the dam at the point of entering the water treatment plants before passing through the plant. Treated water samples were taken from the final water after passing through all purification stages from the treatment plants before pumping to the municipal taps for public consumption while samples from the municipal taps were taking from the taps at the point of public consumption.

### Extraction of DNA and molecular characterization of bacteria using 16S rDNA

The stock culture was streaked on Luria Betani (LB) agar and grown overnight. Afterwards, total genomic DNA was extracted by dispensing 200 µl of 5 % chelex in a tube. A loopful of the overnight grown colony of bacterium from LB agar was then inoculated into the solution. The mixture was then boiled at 100 °C for 10 min and centrifuge at 13 k×*g* for 1 min. Five microliter of the supernatant which consist of the extracted DNA was then used as template with 2 mM MgCl_2_, 0.8 mM dNTPs, 0.2 µl of primer 1 and primer 2 and 1× PCR buffer. The reaction condition include 1 min denaturation (95 °C) followed by 30 cycles of 96 °C for 30 s, 60 °C for 30 s and 72 °C for 30 s and a final extension of 72 °C for 10 min. PCR products were then separated and visualized on 1 % agarose gel electrophoresis to confirm amplification. The 16S rDNA sequence was amplified using 16s-8F (AGAGTTTGATCMTGGCTCAG) and 16s-517 (ATTACCGCGGCTGCTGG) primers. PCR products were sequenced (Eurofins MWG, USA) and manual base calls and sequence trimming was completed by sequencer (5.0) BLAST [12] was used to identify close sequence matches (http://www.ncbi.nlm.nib.gov/BLAST/blast).

### Phylogenetic tree construction

Phylogenetic relatedness of the 16s rDNA genes of these *Pseudomonas* was compared with 12 downloaded 16s rDNA sequences of other species from the gene bank. Sequences were aligned by multiple sequence alignment technique using CLUSTAL W [13] and a phylogenetic tree constructed by the neighbor joining method [14] using MEGA version 5 [15]. *Azotobacterchroococcum* (JQ692178)’s 16s rDNA was used as an outgroup.

### Antimicrobial drug susceptibility and selection of multi-drug resistant *Pseudomonas* spp

Multi-drug resistant *Pseudomonas* was selected based on their resistance to over three classes of antibiotics. This was carried out after assessing antibiotic resistant properties of the *Pseudomonas* using break point assays on LB agar plates [9]. This involves autoclaving of LB agar and cooling down to 45 °C in a water bath and seeding with antibiotics of specific concentration (Table 1) based on Clinical Laboratory Standards Institute (CLSI) standard for gram negative bacteria [16]. Afterwards, the medium was then poured into Petri dishes (150 × 15 mm) and allowed to set. Overnight culture were then stabbed from 96-well plate onto agar plate using 96-well pin replicator and incubated overnight at 37 °C. Isolates were scored as ‘1’ for growth and ‘0’ for no growth on each antibiotics plate. It should be noted that *E. coli* strain K12 was used as negative control while *E. coli* strain H5N was used as positive control.

**Table 1:** Antibiotic concentrations tested against *Pseudomonas* spp

Code	Name	Concentration (µg/ml)
FF	Florfenicol	16
T	Tetracycline	16
S	Streptomycin	16
G	Gentamycin	16
K	Kanamycin	64
C	Chloramphenicol	32
N	Nalidixic acid	30
AMC	Amoxicillin/clavulanic acid	32/16
CEF	Ceftiofur	12
SU	Sulfamethoxazole	512
SXT	Sulfamethoxazole/trimethoprim	76/4

### Assessing antibiotic resistance genes

In this study, six tetracycline, three sulfonamide, three extended β-lactamase and three streptomycin/spectinomycin resistance genes were amplified using specific primer specific for each gene and the annealing temperature as described in Table 2. The amplification of all the genes was carried out using 5 µl of the chelex extracted DNA as template for the PCR mixture with 5 µl of PCR buffer (1×), 2 µl of MgCl_2_ (2 mM), 1 µl of dNTPs 0.8 mM and 1 µl (0.2 µl) each of the forward and reverse primers in a thermo cycler (Model: Bio Rad Laboratories, Richmond, CA, USA). The condition for the amplification of the genes include: 1 min of denaturation of 95 °C followed by 30 cycles of 96 °C for 30 s 60 °C for 30 s and 72 °C for 30 s and final extension of 72 °C for 10 min. All these conditions remain the same during the amplification of the genes except the annealing temperature which is described in Table 2. PCR products were then separated and visualized on 1 % agarose gel electrophoresis to confirm amplification.

**Table 2:** Primers used in this study for amplification of class 1 and class 2 integrons and other antibiotic resistant genes

Primer pair	Target	Sequence (5´–3´)	Annealing temperature (◦C)	Amplicon size (bp)	References
Primer 1 16s-8F	16S rDNA	AGAGTTTGATCMTGGCTCAG	60	456	[36]
Primer 2 16s-517	16S rDNA	ATTACCGCGGCTGCTGG	60	456	[37]
IntI1_F	Class 1 integrase gene	CCTCCCGCACGATGATC	55	270	[38]
IntI1_R		TCCACGCATCGTCAGGC			
IntI2_F	Class 2 integrase gene	TTATTGCTGGGATTAGGC	50	233	[39]
IntI2_R		ACGGCTACCCTCTGTTATC			
5′_CS	Class 1 integron variable region	GGCATCCAAGCAGCAAG	58.5	Variable	[40]
3′_CS		AAGCAGACTTGACCTGA			
Hep_F	Class 2 integron variable region	CGGGATCCCGGACGGCATGCACGATTTGTA	60	Variable	[41]
Hep_R		GATGCCATCGCAAGTACGAG			
Sul1-F	*sul*1	CGGCGTGGGCTACCTGAACG	60	433	[42]
Sul1-R		GCCGATCGCGTGAAGTTCCG			
Sul2-F	*sul*2	GCGCTCAAGGCAGATGGCATT	60	293	[42]
Sul2-R		GCGTTTGATACCGGCACCCGT			
pVP440sul3F	*sul*3	TCAAAGCAAAATGATATGAGC	55	787	[42]
pVP440sul3R		TTTCAAGGCATCTGATAAAGAC			
qacEΔ1F	*qacE*Δ1F	ATC GCA ATA GTT GGC GAA GT	58	800	[43]
sul1-B	*sul*1-B	GCA AGG CGG AAA CCC GCG CC	58		[44]
aph (3″)^c^-F	*aph* (3″)^c^	GCTCAAAGGTCGAGGTGTGG	55	515	[27]
aph (3″)^c^-R		CCAGTTCTCTTCGGCGTTAG	55	515	[27]
ant (3″)^b^-F	*ant* (3″)^b^	CAGCGCAATGACATTCTTGC	55	295	[27]
ant (3″)^b^-F		GTCGGCAGCGACA(C/T)CCTTCG	55	295	[27]
aph(6)-1d^d^-F	*aph*(6)-1d^d^	GACTCCTGCAATCGTCAAGG	55	560	[27]
aph(6)-1d^d^-R		GCAATGCGTCTAGGATCGAG	55	560	[27]
tet(A)-F	*tet*(A)	TTGGCATTCTGCATTCACTC	60	494	[45]
tet(A)-R		GTATAGCTTGCCGGAAGTCG	60	494	[45]
tet(B)-F	*tet*(B)	CAGTGCTGTTGTTGTCATTAA	60	571	[45]
tet(B)-R		GCTTGGAATACTGAGTGTAA	60	571	[45]
tet(E)-F	*tet*(E)	TATTAACGGGCTGGCATTTC	55	544	[45]
tet(E)-R		AGCTGTCAGGTGGGTCAAAC	55	544	[45]
tet(M)-F		ACACGCCAGGACATATGGAT	55	536	[45]
tet(M)-R	*tet*(M)	ATTTCCGCAAAGTTCAGACG	55	536	[45]
tet(30)-F	*tet*(30)	CCGTCATGCAATTTGTGTTC	55	550	[45]
tet(30)-R		TAGAGCACCCAGATCGTTCC	55	550	[45]
SHV_F	*bla*_SHV_	GCGAAAGCCAGCTGTCGGGC	62	538	[46]
SHV_R		GATTGGCGGCGCTGTTATCGC	62	538	[46]
CTX_F	*bla*_CTX-M_	GTGCAGTACCAGTAAAGTTATGG	55	538	[46]
CTX_R		CGCAATATCATTGGTGGTGCC		538	[46]
TEM_F	*bla*_TEM_	AAAGATGCTGAAGATCA	44	425	[46]
TEM_R		TTTGGTATGGCTTCATTC		425	[46]

### Assessing class 1 and 2 integrons

The integrase genes of class 1 and class 2 were amplified by the use of PCR using 5 µl of DNA template extracted by chelex method described above as template. Primer specific for the integrase gene and variable region was selected as described on Table 2. Condition for the PCR was also as described above except for the annealing temperature which was as described on Table 2. It should be noted that after amplification of gene cassette of both Class 1 and Class 2 integrons using 5′-CS/3′-CS and hep_F/hep_R, respectively. PCR products of the amplified genes were used for sequencing (Eurofins MWG, USA). The putative gene cassettes harboured on the integrase sequences were determined through online similarity searches using the BLAST software in the National Center for Biotechnology Information website (http://www.ncbi.nlm.nih.gov/).

### Determination of presence of plasmid on bacteria isolates

Bacteria (*Pseudomonas* isolates) were grown on Luria–Bertani (LB) agar overnight and a single colony was picked and inoculated overnight as well in LB Broth. One hundred and fifty microliter of the culture was later pelleted from the culture by centrifugation for 10 min at 1000×*g*. The supernatant was removed and re-suspended in 100 µl of lysis buffer (3 % SDS, 50 mM Tris pH 12.6 with 50 mM Tris adjusted by 1.6 ml 2 N NaOH up to 100 ml final volume). The mixture was incubated at 55 °C for 1 h. The resulting plasmid was extracted with 150 µl of phenol:chloroform (1:1, v/v, pH 7.9) and mixed by inversion several times before spinning at highest speed for 10 min. Fifty microliter of the supernatant was transferred into a new tube and mixed with 10 µl of 10× loading dye (Invitrogen). The mixture containing the plasmid was run on 1 % agarose gel electrophoresis in 1× TAE (tris-acetate buffer).

## Results

### Bacteria isolates

**Fig. 1:**
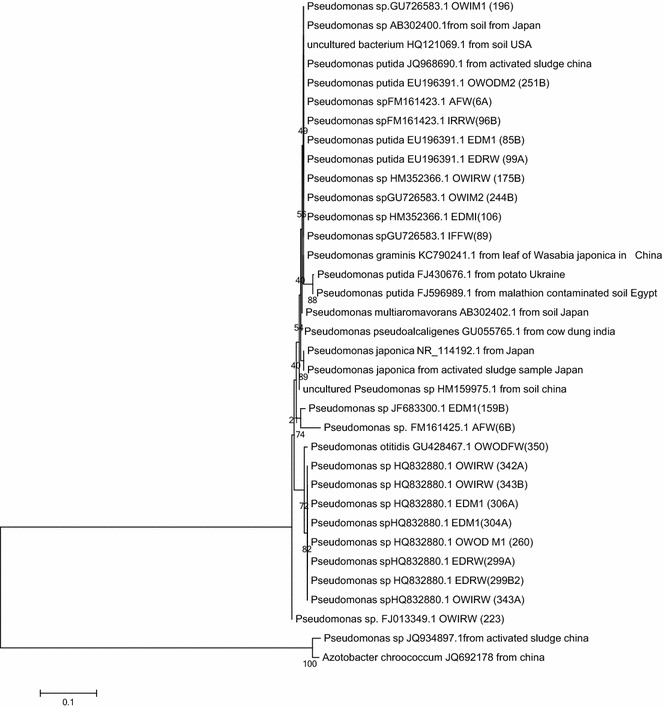
Phylogenetic relationship of 16S rDNA of *Pseudomonas* spp from south western Nigeria water distribution system with sequence from gene bank

In this study, 22 MDR *Pseudomonas* were identified by 16S rDNA out of a total of 296 different strains that were isolated from all sampled water as *P. putida*, *P.fluorescens* and *P.otitidis* (Table 3). Phylogenetic studies showed 10 of the Pseudomonads to be in single distinct cluster with *P. graminis* (KC790241.1) from leaf of *Wasabia japonica* from China, *P. putida* (JQ968690.1) from activated sludge in China, *Pseudomonas* spp (AB302400.1) from Japan and uncultured bacterium (HQ121069.1) from USA. Others such as *Pseudomonas* spp (JF683300) and *Pseudomonas* spp (FM161425.1) from Ede municipal tap and Asejire treated water, respectively belong to the same cluster (Fig. 1).

**Table 3:** Phenotypes, antibiotics resistance gene and integrons among MDR *Pseudomonas* *spp*

Source	Strain ID	Bacteria	Phenotype	*tet*(A)	*tet* (B)	*tet* (E)	*tet*(M)	*tet* (30)	*Sul 1*	*Sul 2*	*bla*SHV	*bla*CTX	*bla*TEM	*aph* (*3″*)^*c*^	*ant* (*3″*)^*b*^	*aph* (*6*)*1d* ^*d*^	int 1	v 1	int 2	v 2
IRW	96b	*P.* *putida*	FF, T, CEF, SU, AMC	−	−	−	−	−	−	−	−	−	−	−	−	−	−	−	−	−
IFW	89	*P. putida*	T, S, G, K, C, SXT, AMC, SU	+	−	−	−	+	−	−	+	−	+	+	+	−	−	−	−	−
EDRW	299A	*P.* *otitidis*	FF, C, SXT, AMC, SU	−	−	−	−	−	−	−	−	−	−	−	−	−	−	−	−	−
EDRW	99A	*P. putida*	FF, SXT, AMC	−	−	−	−	−	−	+	−	−	−	−	−	−	−	−	−	−
EDRW	299B2	*P.* *otitidis*	FF, CEF, AMC, SU	−	−	−	−	−	−	−	−	−	−	−	+	−	−	−	−	−
EDM1	159B	*P. fluorescens*	T, S, G, N, CEF, SXT	−	−	−	−	−	−	−	−	−	+	−	−	−	−	−	−	−
EDM1	304A	*P.* *otitidis*	T, SU, SXT, AMC, S, G, CEF, FF	−	−	−	−	−	−	−	−	−	+	−	+	−	−	−	−	−
EDM1	306A	*P.* *otitidis*	FF, CEF, AMC, SU	−	−	−	−	−	−	−	−	−	−	−	−	−	−	−	−	−
EDM1	85B	*P. putida*	SXT, AMC, C, FF	−	−	−	−	−	−	+	+	−	−	−	−	−	−	−	−	−
EDM1	106	*P.* *putida*	T, SU, SXT, AMC, S, N	−	−	−	−	−	−	−	−	−	+	−	−	−	−	−	−	−
AFW	6B	*P. fluorescens*	T, S, G, K, CEF, SXT	−	−	−	−	−	−	+	−	−	−	−	−	−	−	−	−	−
AFW	6A	*P. fluorescens*	T, S, G, K, C, CEF, SXT, AMC, SU, FF	+	−	−	−	−	+	−	+	−	+	+	+	−	+	+	−	−
OWODFW	350	*P. otitidis*	SXT, SU	−	−	−	−	−	+	−	−	−	−	−	−	−	−	−	−	−
OWODM1	260	*P.* *putida*	FF, T, C, N, CEF, SXT, AMC, SU	−	−	−	−	−	−	−	−	−	+	−	+	−	−	−	−	−
OWODM2	251B	*P. putida*	T, S, C, SXT, SU	+	−	−	−	−	+	+	+	−	−	+	+	+	+	+	−	−
OWIRW	343B	*P.* *otitidis*	FF, T, S, N, CEF, AMC, SU	+	−	−	−	−	+	−	−	−	−	−	−	−	+	−	−	−
OWIRW	343A	*P.* *otitidis*	FF, K, C, SXT, N, CEF, AMC, SU	−	−	−	−	−	−	−	−	−	−	+	−	−	−	−	−	−
OWIRW	223	*P.* *otitidis*	FF, S, C, CEF, SXT, AMC, SU	−	−	−	−	−	−	−	−	−	−	+	−	−	−	−	−	−
OWRW	175B	*P.* *putida*	T, S, C, K, CEF, SXT, AMC, SU	+	−	−	−	−	+	+	−	−	+	+	−	+	+	+	−	−
OWIRW	342A	*P.* *otitidis*	FF, AMC, CEF, SU	−	−	−	−	−	−	−	−	−	−	−	−	−	−	−	−	−
OWIM1	196	*P.* *putida*	FF, T, S, G, K, AMC, SU	+	−	+	−	−	+	−	−	−	+	+	+	−	+	+	−	−
OWIM2	244B	*P.* *putida*	FF, T, S, G, K, C, SXT, AMC, SU	+	−	−	−	−	+	+	+	−	+	+	+	+	+	+	−	−
			Total	7	0	1	0	1	7	6	5	0	9	8	8	3	6			
			Percentages	31.8	0	4.6	0	4.6	31.8	27.3	27.3	0	40.9	36.4	36.4	13.6	27.3			

Antibiotics code: *FF* florfenicol, *T* tetracycline, *S* streptomycin, *G* gentamicin, *K* kanamycin, *C* chloramphenicol, *N* nalidixic acid, *AMC* amoxicillin/clavulanic acid, *CEF* ceftiofur, *SU* sulfamethoxazole, *SXT* sulfamethoxazole/trimethoprim

Source code: *IFW* Ife treated water, *EDM1* Ede municipal Tap 1, *AFW* Asejire treated water, *OWODM2* Owena Ondo Municipal Tap 2, *OWIRW* Owena-Idanre raw water, *OWIM1* Owena-Idanre Municipal Tap 1, *OWIM2* Owena-Idanre Municipal Tap 2

### Antimicrobial susceptibility

**Fig. 2:**
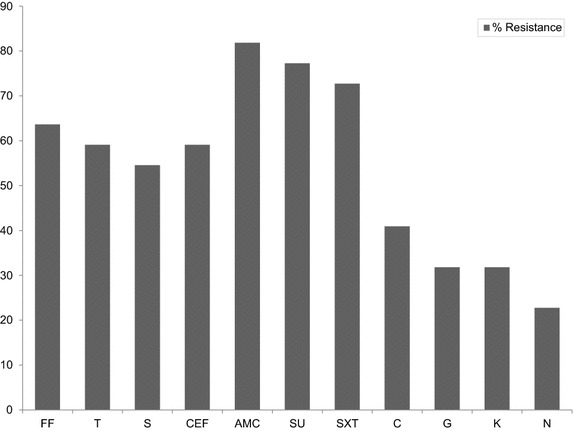
Percentage resistance of *Pseudomonas* spp to various antibiotics. *FF* florfenicol, *T* tetracycline, *S* streptomycin, *G* gentamicin, *K* kanamycin, *C* chloramphenicol, *N* nalidixic acid, *AMC* amoxicillin/clavulanic acid, *CEF* ceftiofur, *SU* sulfamethoxazole, *SXT* sulfamethoxazole/trimethoprim

Figure 2 and Table 3 showed the results of the percentage resistance of the studies bacteria and their phenotypic patterns of resistance, respectively. We observed among the studied *Pseudomonas* higher resistance to florfenicol (63.6 %), tetracycline (59.1 %), streptomycin (54.6 %), ceftiofur (59.1 %), amoxicillin/clavulanic acid (81.8 %), sulfamethoxazole (77.3 %) compared to chloramphenicol (40.9 %), gentamicin (31.8 %), kanamycin (31.8 %) and nalidixic acid (22.7 %). From these results, it was noticed that these isolates were more resistant to older generation antibiotics (such as sulfamethoxazole, tetracycline, streptomycin etc.) than new generation antibiotics such as (gentamicin, kanamycin and nalidixic acid). We also saw that out of 22 *Pseudomonas* spp isolated from all the sampled area 21 were multi-drug resistant (MDR) (Table 3). The MDR bacteria were selected based on resistant to over three classes of antibiotics.

### Molecular characterization of antibiotic resistance genes

Beyond the phenotypic determination, we tested for the presence of some antibiotic resistance genes which could have been mediating the antibiotic resistance in the *Pseudomonas* spp. Among the tetracycline resistance gene tested i.e. *tet*(A), *tet*(B), *tet*(E) and *tet*30 encoding resistant by efflux pump mechanism and *tet*(O) and *tet*(M) encoding resistant by protection of the ribosome. We observed that *tet*(A) was the highest occurred (31.8 %) followed by *tet*(E) and *tet*30 (4.5 %) (Table 3). We did not detect *tet*(B), *tet*(M) and *tet*(O) among the *Pseudomonas* isolates. Among three sulphonamide resistance genes tested for in this study. It was observed that *sul 1* was the highest (31.8 %) detected followed by *sul 2* (27.3 %) (Table 3). *Sul 3* was not detected in any of the *Pseudomonas* while among the three extended beta lactamase (ESBL) genes: *bla*_SHV_, *bla*_CTX_, *bla*_TEM_, we observed that *bla*_TEM_ was the highest (40.9 %) detected followed by *bla*_SHV_ (27.3 %) while *bla*_CTX_ was not detected at all (Table 3).

We also observed the presence of all three streptomycin resistance genes tested for in at least one of the isolates (Table 3). *Aph*(*3″*)^*c*^ and *ant*(*3″*) was the most detected, each was observed to be present in 36.4 % of isolates tested while *aph* (*6*)*1d*^*d*^ was the least (13.7) detected. Class 2 integron was not detected in any of the isolates (Table 3). Whereas, 6 (27.3 %) of the isolates showed the occurrence of class 1 integron.

### Plasmid profiling

The presence of plasmid was observed in 10 (45.5 %) out of 22 *Pseudomonas* studied (Table 4). Each of the bacteria was observed to be carrying 1 plasmid each ranging between 22 and 130 kb in size. None of the bacteria showed the occurrence of more than one plasmid.

**Table 4:** Plasmid carrying *Pseudomonas* spp. isolated from selected water samples from southwestern Nigeria

Source	Bacteria/strain ID	Resistant phenotypes	No of plasmid and size
IFW	*Pseudomonas* sp (89)	T, S, G, K, C, SXT, AMC, SU	1 (95 kb)
EDM1	*Pseudomonas* sp (306A)	FF, CEF, AMC, SU	1 (95 kb)
AFW	*Pseudomonas* sp (6A)	T, S, G, K, C, CEF, SXT, AMC, SU, FF	1 (95 kb)
AFW	*Pseudomonas* sp (6B)	T, S, G, K, CEF, SXT	1 (95 kb)
OWODM2	*Pseudomonas putida* (251B)	T, S, C, SXT, SU	1 (22 kb)
OWIRW	*Pseudomonas* sp (175B)	T, S, C, K, CEF, SXT, AMC, SU	1 (55 kb)
OWIRW	*Pseudomonas* sp (342A)	FF, AMC, CEF, SU	1 (130 kb)
OWIRW	*Pseudomonas* sp (343B)	FF, T, S, N, CEF, AM, AMC, SU	1 (130 kb)
OWIM1	*Pseudomonas* sp (196)	FF, T, S, G, K, AM, AMC, SU	1 (120 kb)
OWIM2	*Pseudomonas* sp (244B)	FF, T, S, G, K, C, AM, SXT, AMC, SU	1 (130 kb)

Antibiotics code: *FF* florfenicol, *T* tetracycline, *S* streptomycin, *G* gentamicin, *K* kanamycin, *C* chloramphenicol, *N* nalidixic acid, *AMC* amoxicillin/clavulanic acid, *CEF* ceftiofur, *SU* sulfamethoxazole, *SXT* sulfamethoxazole/trimethoprim

Source code: *IFW* Ife treated water, *EDM1* Ede municipal Tap 1, *AFW* Asejire treated water, *OWODM2* Owena Ondo Municipal Tap 2, *OWIRW* Owena-Idanre raw water, *OWIM1* Owena-Idanre Municipal Tap 1, *OWIM2* Owena-Idanre Municipal Tap 2

## Discussion

Pseudomonad is one of the most important and ubiquitous pathogen that is significantly important medically [17]. Aquatic ecosystems could serve as a reservoir of antibiotic resistant pathogen through transfer of resistant plasmid [18]. Many studies on antibiotic resistance in pseudomonads had been carried out using strains of clinical origin [19]. This study selected 22 MDR Pseudomonas from drinking water distribution systems from southwestern Nigeria. These selected MDR strains showed high resistant to amoxillin/clavulanic acid (81.8 %), sulfamethoxazole (77.3 %) and sulfamethoxazole/trimethoprim (72.7 %). Resistant to amoxillin/clavulanic acid is a little similar to the report of Mezue et al. [20] among *Pseudomonas* from urinary tract infection from Nigeria who reported 100 % resistant to the antibiotics in their studies. Moderate resistant to florfenicol (63.6 %), tetracycline (59.1 %), streptomycin (54.6 %) and ceftiofur (59.1 %) was observed among these Pseudomonads. The tetracycline resistance obtained in our study is similar to what Akinpelu et al. [21] reported among *Pseudomonas* from another river in Nigeria. The author reported 50 % resistant to tetracycline among their pseudomonads while higher (100 %) resistant to streptomycin compare to what we obtained from the isolates in this study.

Molecular characterization of the antibiotic resistance gene showed high prevalence of *tet*(A) (31.8 %), *sul2* (27.8 %), *bla*_TEM_ (40.9) for tetracycline, sulfa drug and β-lactamase antibiotic resistance, respectively. There appears to be few studies on the molecular characterization of these genes among Nigeria environmental pseudomonads, therefore, making it difficult to compare with studies from Nigeria. However, among few of the studies, Chikwendu et al. [22] reported the presence of *bla*_SHV_ and *bla*_TEM_ among environmental pseudomonads from Nigeria while Odumosu et al. [23] reported *bla*_oxa-10_, ampC β-lactamase in 50 and 70 % of *Pseudomonasaeruginosa*, respectively from Nigeria clinical source. The authors reported that only one of their isolates testing positive for *bla*_SHV_ and *bla*_CTXM-15_. Studies have shown that due to overuse of β-lactam antibiotics, *bla*_TEM_ and *bla*_SHV_ enzyme that were originally found in enterobacteriaceae are now found in *Pseudomonas* [24]. Similarly, the authors also reported that *bla*_SHV_ enzyme that has been found in *Klebsiella*, *Escherichia* and *Salmonella* can also be found in *Pseudomonas*. Moreover, in this study we did not detect *bla*_TEM_ from any of the *Pseudomonas* studied from these water distribution systems.

We observed 36.36 % of our pseudomonas testing positive for each of *aph*(*3″*)^*c*^ and *ant*(*3″*)^*b*^ which are genes coding for enzymes that modifies aminoglycoside antibiotics. Odumosu et al. [23] also reported the presence of aminoglycoside modifying enzyme (AME) genes which include *aac*(*6′*)-*I* and *ant*(*2″*)-*I* in 50 and 45 %, respectively of their isolates from Nigeria clinical *Pseudomonas*. Publications on AME genes among Nigeria environmental *Pseudomonas* spp are also scarce. This could be the first time these genes will be described among pseudomonad from Nigeria environment. However, in Africa, Ndegwa et al. [25] reported the presence of AME in *E. coli*, *klebsiella*, *Pseudomonas* and *Acinetobacter* from clinical isolates from Kenya. Though in this study, at least one of the AME genes occurred in at least one of these environmental bacteria. It has been reported that the incidence of streptomycin resistance in pathogenic and commensal bacteria in clinical and agricultural habitats is often high. This is most likely caused by the use of streptomycin in these environments [26]. We observed *aph* (*3″*)^*c*^ and *ant* (*3″*) to be more frequently detected in this study than *aph* (*6*)*1d*^*d*^. This is similar to the study of van Overbeek et al. [27] among bacterial populations in European habitat. The authors reported that out of 22 bacteria isolates whose genomic DNA was extracted and amplified for streptomycin resistance gene, *aph* (*3″*)^*c*^ was the highest occurred, showing its presence in 17 isolates, followed by *ant* (*3″*) and *aph* (*6*)-*1d* (both present in 13 isolates), whereas the incidence of *aph* (*6*)-*1c* and *ant* (*6*) genes was lowest (present in one and zero isolates, respectively). Occurrence of one bacteria carrying more than one of streptomycin resistance genes was observed in this study. This is also similar to the report of van Overbeek et al. [27].

Similarly, tetracycline resistance gene has also not been described in among Pseudomonads from Nigeria. Although our recent report [28] stated the occurrence of *tet*(E), *tet*(M), *tet*(30) among *Alcaligenes*, *tet*(E) in *Aeromonas* and *Klebsiella*, *tet*(B) in *Bacillus* and *Leucobacter* from these water distribution systems. Absence of *tet*(B) but highest occurrence of *tet*(A) which are both efflux pump genes in these pseudomonads is contrary to the report of Shababi et al. [29] who reported more prevalence of *tet*(B) than *tet*(A) in their studies among MDR *P. aeruginosa* from municipal sewage. However, a study had shown that *tet*(A) has a broad host range and is often carried by various environmental genera [4]. None of the isolates showed the presence of tetracycline resistance gene [*tet*(O) and *tet*(M)] that cause resistant by producing protein that modifies the ribosome.

Occurrence of class 1 integron among these bacteria showed their potential ability to shuttle antibiotic resistance between different bacteria species. This is because integrons are mobile DNA element with the ability to integrate and express gene cassettes by site-specific recombination [30, 31]. Integrase sequence can insert many cassettes coding for different antibiotic resistance, therefore, making a single bacterium possessing it to be resistant to multiple antibiotics. Therefore, emergence of these MDR bacteria with class 1 integron could lead to reduce antibiotic treatment option which could result to an increase possibility of treatment failure, if they infect user of these water. In fact, most of the genes like aminoglycoside resistance, β-lactam resistance and sulpha-drug resistance have been reported as gene cassettes in association with class 1 integron [32]. Five of these pseudomonads were observed to test positive for the variable region of this class 1 integron. This implies the insertion of antibiotic resistant cassette in the integrase sequence and these resistance gene cassettes include aminoglycosides (*aadA2*, *aadA1*), trimethoprim (*dfrA15*, *dfr7*) and sulphonamide (*sul1*) resistance.

The size of plasmids observed from our study contradicts previous studies from Nigeria [6, 33] which reported low molecular weight plasmids (<2 kb) among bacteria from clinical isolates of *P. aeroginosa.* The relationship between plasmid profiles and MDR patterns observed in this study suggests that plasmids may have great role to play in the multidrug resistance of the *Pseudomonas.* Plasmids usually evolve as an integral part of the bacterial genome, consisting of several extra-chromosomal traits, one of which is their resistance genes, which can be exchanged among bacteria of different origins by conjugation [34]. Global dissemination of multiple antibiotic resistance and virulence traits by plasmids also poses an increasing threat to the successful treatment of bacterial infectious diseases in animals and humans alike [35]. Therefore, water sample containing these bacteria may be a source of transfer of antibiotic resistance genes to pathogenic bacteria of human, animal and environmental health significance.

## Conclusion

In summary, the occurrence of MDR *Pseudomonas* harbouring various antibiotic resistance genes, class 1 integron and plasmid in drinking water systems signify that these environmental pseudomonads could significantly contribute to wide-scale transfer of resistance genes in these environments thereby posing health hazard to man and animals that depend on these water sources. This could play a major role in the epidemiology of antibiotic resistance in Nigeria and could also be responsible for prolong treatment of bacterial infection, the emergence of emerging and re-emerging infectious diseases. We hereby recommend integrated antibiotic resistant surveillance system and holistic one health approach as well as promoting prudent use of antibiotics both in clinical and agriculture settings in order to prevent and control multidrug resistant bacteria in southwestern Nigeria.

## References

[1] Craun GF, Calderon RL, Craun MF (2005). Outbreaks associated with recreational water in the United States. Int J Environ Health Res.

[2] Pollack M. Principles and practice of infectious diseases, eds. In: Mandell, G. L., Bennet, J. E. and Dolin, R. (*Churchill Livingstone*, *Philadelphia*).2000 2: 2310-2335.

[3] Lyczak JB, Cannon CL, Pier GB (2002). Lung infection associated with cystic fibrosis. Clin Microbiol Rev.

[4] Zhang XX, Zhang T, Fang HHP (2009). Antibiotic resistance genes in water environment. Appl Microbiol Biotechnol.

[5] Bonomo RA, Szabo D (2006). Mechanisms of multidrug resistance in *Acinetobacter* species and *Pseudomonas**aeruginosa*. Clin Infect Dis.

[6] Yah SC, Eghafona NO, Enabulele IO (2006). Prevalence of plasmids mediated *Pseudomonas**aeruginosa* resistant genes from burn wound patients at the university of Benin teaching hospital Benin City. Nigeria J Biomed Sci.

[7] Chen J, Su Z, Liu Y, Wang S, Dai X, Li Y, Peng S, Shao Q, Zhang H, Wen P, Yu J, Huang X, Xu H (2009). Identification and characterization of class 1 integrons among *Pseudomonas**aeruginosa* isolates from patients in Zhenjiang, China. Int J Infect Dis.

[8] Ruiz-Martínez L, López-Jiménez L, Fusté E, Vinuesa T, Martínez JP, Viñas M (2011). Class integrons in environmental and clinical isolates of *Pseudomonas**aeruginosa*. Int J Antimicrob Agents.

[9] Adesoji AT, Ogunjobi AA (2013). Occurrence of multidrug-resistant bacteria in selected water distribution systems in Oyo State, Nigeria. Glob Vet.

[10] Adesoji AT, Ogunjobi AA (2013). Physicochemical properties and occurrence of antibiotic-resistant bacteria in Ife and Ede water distribution systems of southwestern Nigeria. World Appl Sci J.

[11] Adesoji AT, Ogunjobi AA, Olatoye IO (2014). Drinking water distribution systems of dams in Ondo state, Nigeria as reservoir of multi-drug resistant bacteria. World Appl Sci J.

[12] Altschul SF, Madden TL, Schäffer AA, Zhang J, Zhang Z, Miller W, Lipman DJ. Gapped BLAST and PSI-BLAST: a new generation of protein database search programs. Nucleic Acids Res. 2007;25:3389–402. http://www.ncbi.nlm.nih.gov/BLAST. 10.1093/nar/25.17.3389 PMC146917 9254694

[13] Thompson JD, Higgins DG, Gibson TJ (1994). CLUSTAL W: improving the sensitivity of progressive multiple sequence alignment through sequence weighting, position-specific gap penalties and weight matrix choice. Nucleic Acids Res.

[14] Saitou N, Nei M (1987). The neighbor-joining method: a new method for reconstructing phylogenetic trees. Mol Bio Evol.

[15] Kumar S, Tamura K, Nei M (2004). MEGA3: integrated software for molecular evolutionary genetics analysis and sequence alignment. Brief Bioinform.

[16] CLSI (Clinical and Laboratory Standards Institute) Performance standards for antimicrobial susceptibility testing: 15th informational supplement M100-S15. Wayne: CLSI; 2005.

[17] Pollack M, Mandell GL, Dolan R, Bennett JE (1995). *Pseudomonas aeruginosa*. Principles and practices of infectious diseases.

[18] Baquero F, Martínez JL, Cantó N (2008). Antibiotics and antibiotic resistance in water environments. Curr Opin Biotechnol.

[19] Ndip RN, Dilonga HM, Ndip LM, Akoachere JF, Nkuo Akenji T (2005). *Pseudomonas**aeruginosa* isolates recovered from clinical and environmental samples in Buea, Cameroon: current status on biotyping and antibiogram. Trop Med Int Health.

[20] Mezue K, Ofong C, Nmezi D, Ugochukwu-Obi G. Antibiotic Sensitivity patterns in urinary tract infection at Tertiary Hospital. Medikka J Univ Nigeria Med Stud. 2006. Available on http://www.medikkajournal.com/UTI.htm. Extracted on 7/01/2015.

[21] Akinpelu AT, Akinloye OM, Olukemi BC, Adegoke AE, Olayinka S (2014). Antibiotic resistant pattern of isolated bacteria from Obere River in Orile-Igbon, Oyo State Nigeria. Afr J Microbiol Res.

[22] Chikwendu CI, Ibe SN, Okpokwasili GC (2011). Detection of *bla*_SHV_ and *bla*_TEM_ beta-lactamase genes in multi-resistant *Pseudomonas* isolates from environmental sources. Afr J Microbiol Res.

[23] Odumosu BT, Adeniyi BA, Chandra R (2013). Analysis of integrons and associated gene cassettes in clinical isolates of multidrug resistant *Pseudomonas**aeruginosa* from Southwest Nigeria. Ann Clin Microbiol Antimicrob.

[24] Bradford PA (2001). Extended-spectrum beta-lactamases in the 21st century: characterization, epidemiology, and detection of this important resistance threat. Clin Microbiol Rev.

[25] Ndegwa DW, Budambula NLM, Kariuki G, Kiiru JN. Aminoglycoside modifying enzymes detected in strains of *Escherichia*, *Klebsiella*, *Pseudomonas* and *Acinetobacter* implicated in invasive infections in Nairobi, Kenya. In: Proceedings of 2010 JKUAT scientific technological and industrialization conference Centre for Microbiology. Nairobi, Kenya: Kenya Medical Research Institute; 2010.

[26] Seveno NA, Kallifidas D, Smalla K, van Elsas JD, Collard JM, Karagouni AD, Wellington EMH (2002). Occurrence of reservoirs of antibiotic resistance genes in the environment. Rev Med Microbiol.

[27] van Overbeek LSV, Wellington EMH, Egan S, Smalla K, Heuer H, Collard JM, Guillaume G, Karagouni AD, Nikolakopoulou TL, Elsas JDVE (2001). Prevalence of streptomycin-resistance genes in bacterial populations in European habitats. FEMS Microbiol Ecol.

[28] Adesoji AT, Ogunjobi AA, Olatoye IO, Call DR (2015). Prevalence of tetracycline resistance genes among multi-drug resistant bacteria from selected water distribution systems in southwestern Nigeria. Ann Clin Microbiol Antimicrob..

[29] Shehabi AA, Haider AA, Fayyad MK (2011). Frequency of antimicrobial resistance markers among *Pseudomonas**aeruginosa* and *Escherichia**coli* isolates from municipal sewage effluent water and patients in Jordan. Int Arabic J Antimicrob Agents.

[30] Mazel D (2006). Integrons: agents of bacterial evolution. Nat Rev Microbiol.

[31] Rowe-Magnus DA, Mazel D (2002). The role of integrons in antibiotic resistance gene capture. Int J Med Microbiol.

[32] Poonsuk K, Tribuddharat C, Chuanchuen R (2012). Class 1 integrons in *Pseudomonas aeruginosa* and *Acinetobacter baumannii* isolated from clinical isolates. Southeast Asian J Trop Med Public Health.

[33] Olayinka AT, Onile BA, Olayinka BO (2004). Prevalence of multi-drug resistant (MDR) *Pseudomonas**aeruginosa* isolates in surgical units of Ahmadu Bello University Teaching Hospital, Zaria, Nigeria: an indication for effective control measures. Afr Med.

[34] Carattoli A (2011). Plasmids in Gram negatives: molecular typing of resistance plasmids. Int J Med Microbiol.

[35] Bush K (2010). Bench-to-bedside review: the role of beta-lactamases in antibiotic-resistant Gram-negative infections. Crit Care.

[36] Baker GC, Smith JJ, Cowan DA (2003). Review and reanalysis of domain-specific 16S primers. J Microbiol Methods.

[37] Muyzer G, de Waal EC, Uitterlinden AG (1993). Profiling of complex microbial populations by denaturing gradient gel electrophoresis analysis of polymerase chain reaction-amplified genes coding for 16S rRNA. Appl Environ Microbiol.

[38] Houang AT, Chu YW, Lo WS, Chu KY, Cheng AF (2003). Epidemiology of rifampin ADP-ribosyltransferase (*arr*-*2*) and metallo-b-lactamase (*bla*_*IMP*-*4*_) gene cassettes in class 1 integrons in *Acinetobacter* strains isolated from blood cultures in 1997 to 2000. Antimicrob Agents Chemother.

[39] Roe MT, Vega E, Pillai SD (2003). Antimicrobial resistance markers of Class 1 and Class 2 integron bearing *Escherichia**coli* from irrigation water and sediments. Emerg Infect Dis.

[40] Hall RM, Collis CM (1995). Mobile gene cassettes and integrons: capture and spread of genes by site-specific recombination. Mol Microbiol.

[41] White PA, Mciver CJ, Rawlinson WD (2001). Integrons and gene cassettes in the Enterobacteriaceae. Antimicrob Agents Chemother.

[42] Vinue L, Saenz Y, Rojo-Bezares B, Olarte I, Undabeitia E, Somalo S, Zarazaga M, Torres C (2010). Genetic environment of *sul* genes and characterisation of integrons in *Escherichia coli* isolates of blood origin in a Spanish hospital. Int J Antimicrob Agents.

[43] Stokes HW, Hall RM (1989). A novel family of potentially mobile DNA elements encoding site-specific gene-integration functions: integrons. Mol Microbiol.

[44] Sundstrom L (1998). The potential of integrons and connected programmed rearrangements for mediating horizontal gene transfer. APMIS..

[45] Call DR, Brockman FJ, Chandler DP (2003). Detecting and genotyping *Escherichia coli* O157:H7 using multiplexed PCR and nucleic acid microarrays. Int J Food Microbiol.

[46] Henriques IS, Fonseca F, Alves A, Saavedra MJ, Correia A (2006). Occurrence and diversity of integrons and β-lactamase genes among ampicillin-resistant isolates from estuarine waters. Res Microbiol.

